# Impact of cytotoxic agents or apoptosis stimulants on αklotho in MDCK, NRK-52E and HK2 kidney cells

**DOI:** 10.18632/aging.204238

**Published:** 2022-08-22

**Authors:** Sina Münz, Lisa Wolf, Ludwig E. Hoelzle, Dmitry Chernyakov, Bayram Edemir, Michael Föller

**Affiliations:** 1 Department of Physiology, University of Hohenheim, Stuttgart 70599, Germany; 2 Institute of Animal Science, University of Hohenheim, Stuttgart 70599, Germany; 3 Department of Oncology, Martin-Luther-University Halle-Wittenberg, Halle (Saale) 06120, Germany

**Keywords:** viability, aging, FGF23, cisplatin, doxorubicin

## Abstract

αKlotho is a transmembrane protein acting as a co-receptor for FGF23, a bone hormone regulating renal phosphate and vitamin D metabolism. αKlotho expression is controlled by PPARγ. Soluble αklotho (sKL) regulates cellular signaling impacting stress resistance and death. αKlotho deficiency causes early onset of aging-associated diseases while its overexpression markedly increases lifespan. Cellular stress due to cytotoxic therapeutics or apoptosis induction through caspase activation or serum deficiency may result in cell death. Owing to αklotho’s role in cellular stress and aging, this study explored the effect of cytotoxic agents or apoptosis stimulants on cellular αklotho expression. Experiments were performed in renal MDCK, NRK-52E and HK-2 cells. Gene expression was determined by qRT-PCR, sKL by ELISA, apoptosis and necrosis by annexin V binding and a fluorescent DNA dye, and cell viability by MTT assay. Cytostatic drugs cisplatin, paclitaxel, and doxorubicin as well as apoptosis induction with caspase 3 activator PAC-1 and serum deprivation induced αklotho and *PPARG* gene expression while decreasing viability and proliferation and inducing apoptosis of MDCK and NRK-52E cells to a variable extent. PPARγ antagonism attenuated up-regulation of αklotho in MDCK cells. In HK-2 cells, αklotho gene expression and sKL protein were down-regulated by chemotherapeutics. SKL serum levels in patients following chemotherapy were not significantly changed. In summary, potentially fatal stress results in up-regulation of αKlotho gene expression in MDCK and NRK-52E cells and down-regulation in HK-2 cells. These results indicate that different renal cell lines may exhibit completely different regulation of αklotho.

## INTRODUCTION

The αklotho gene product was discovered in mice in 1997 as a protein with strong anti-aging properties [1, 2]. Mice almost completely lacking αklotho exhibit a dramatically shortened life span of a few weeks only whilst suffering from a broad range of diseases and symptoms mimicking human aging [1]. Observed abnormalities affect nearly every organ and tissue [1] and include frequent aging-associated diseases including fibrosis [3, 4], lung emphysema [5], multiple organ atrophy [1], or hearing loss [6, 7]. The accelerated aging of αklotho-deficient mice is paralleled by massive calcification in most tissues [1, 8]. Importantly, the reduction of dietary phosphate or vitamin D intake of the animals almost completely normalizes their phenotype pointing to a dominant role of phosphate and vitamin D excess in their rapid aging [9, 10]. Indeed, αklotho protein has important functions in the homeostasis of these nutrients [11]: It is a transmembrane protein predominantly expressed in the kidney that enhances the binding affinity of fibroblast growth factor 23 (FGF23) for its membrane receptor [12, 13]. FGF23 is a proteohormone released by bone cells that inhibits phosphate reabsorption and 1,25(OH)_2_D_3_ (biologically active vitamin D) synthesis in the kidney [14, 15] and has gained attention as a marker indicating disease [16, 17]. Hence, the lack of αklotho or FGF23 results in abnormally high serum phosphate and 1,25(OH)_2_D_3_ levels that account for enhanced calcification and contribute to rapid aging and early death to a large extent [18].

In addition to its significance as a co-receptor for FGF23, FGF23-independent endocrine and paracrine effects of αklotho have been revealed [19–21]. These are mainly due to soluble klotho (sKL) that is produced through the cleavage of transmembrane αklotho [22]. SKL can be detected in body fluids including serum, urine, or cerebrospinal fluid [23, 24]. Endocrine or paracrine actions of sKL include the direct regulation of ion channels [25] or important signaling pathways (e.g., IGF, Wnt, or TGF-β1 signaling) [2, 26, 27]. αKlotho exerts anti-neoplastic [28], anti-inflammatory [29, 30], anti-fibrotic [3], and anti-oxidant effects [31, 32] and has been proven organoprotective, e.g., in the kidney [33, 34]. In several tumor cell lines and cancer mouse models, higher expression of αklotho is associated with beneficial, potentially lifespan-expanding effects [35, 36]. And indeed, overexpression of αklotho results in a 30% longer lifespan of mice uncovering αklotho as a very powerful anti-aging factor [2]. Also in human centenarians, single nucleotide polymorphisms (SNPs) of the αklotho gene may be effective [37]. Moreover, lower αklotho levels are associated with poorer outcome in kidney or cardiovascular disease in men [33, 38–40].

Chemotherapy with platinum derivative cisplatin, anthracycline doxorubicin, or paclitaxel is standard of care in many forms of cancer. Although the three compounds differ in their cellular targets, they have in common that they exert cytotoxic effects which compromise proliferation and may ultimately result in apoptotic cell death [41–43]. Apoptosis of cultured cells without prior cell damage may be induced by activation of executioner caspase 3 with PAC-1 or by growth factor deprivation through serum depletion [44, 45].

In view of the versatile effects of αklotho on cell survival and death [46, 47], this study aimed to investigate whether cytotoxic drugs or initiation of apoptosis affect αklotho gene expression in three different renal cell lines and in patients receiving chemotherapy.

## RESULTS AND DISCUSSION

**Figure 1:**
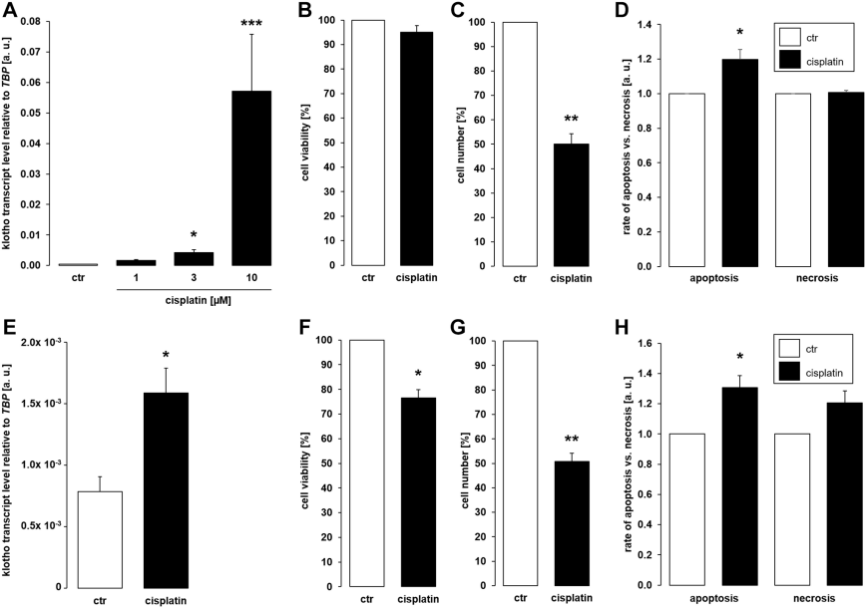
**Cisplatin upregulates αklotho expression in MDCK and NRK-52E cells.** (**A**) Arithmetic mean ± SEM of αklotho transcript levels normalized to *TBP* in MDCK cells treated with cisplatin at the indicated concentration for 24 h (*n* = 5; *Friedman ANOVA* followed by *Dunn-Bonferroni* post-hoc test). (**B**, **C**) Arithmetic mean ± SEM of MDCK cell viability (**B**) or number (**C**) upon treatment without or with 10 μM cisplatin for 24 h (**B**: *n* = 5, *one-sample t-*test; **C**: *n* = 4, *one-sample t-*test). (**D**) Rate of apoptosis and necrosis of MDCK cells treated with or without 10 μM cisplatin for 24 h (*n* = 6, *one-sample t* test) (**E**) Arithmetic mean ± SEM of αklotho transcript levels relative to *TBP* in NRK-52E cells incubated without or with 10 μM cisplatin for 24 h (*n* = 5, *paired t-*test). (**F**, **G**) Arithmetic mean ± SEM of NRK-52E cell viability (**F**) or number (**G**) upon treatment without or with 10 μM cisplatin for 24 h (**F**: *n* = 5, *one-sample t*-test; **G**: *n* = 4, *one-sample t*-test). (**H**) Rate of apoptosis and necrosis of NRK-52E cells treated with or without 10 μM cisplatin for 24 h (*n* = 5, *one-sample t* test) ^*^p < 0,05, ^**^p < 0.01, ^***^p < 0.001 indicate significant difference from vehicle control; Abbreviations: a. u.: arbitrary units; ctr: control.

As a first step, MDCK and NRK-52E cells were used to study αklotho gene expression. MDCK cells were treated with antineoplastic platinum derivative cisplatin for 24 h, and αklotho mRNA levels were analyzed by qRT-PCR. As illustrated in Figure 1A, cisplatin up-regulated αklotho gene expression in MDCK cells, an effect reaching significance at 3 μM cisplatin. The effect was not paralleled by decreased viability of MDCK cells even at 10 μM cisplatin (Figure 1B), but by reduced cell proliferation (Figure 1C). We determined the rate of apoptosis and necrosis by means of an assay analyzing annexin V binding and a DNA-binding dye which is impermeable to the membrane of intact cells. As illustrated in Figure 1D, cisplatin induced apoptosis without significantly influencing necrosis of MDCK cells. In another series of experiments, NRK-52E cells were treated without or with cisplatin for 24 h, and αklotho gene expression, viability, proliferation, and apoptosis/necrosis were assessed. Again, cisplatin (10 μM) significantly enhanced αklotho expression (Figure 1E), an effect paralleled by decreased cell viability (Figure 1F) and proliferation (Figure 1G). Again, cisplatin induced apoptosis without significantly stimulating necrosis of NRK-52E cells (Figure 1H).

**Figure 2:**
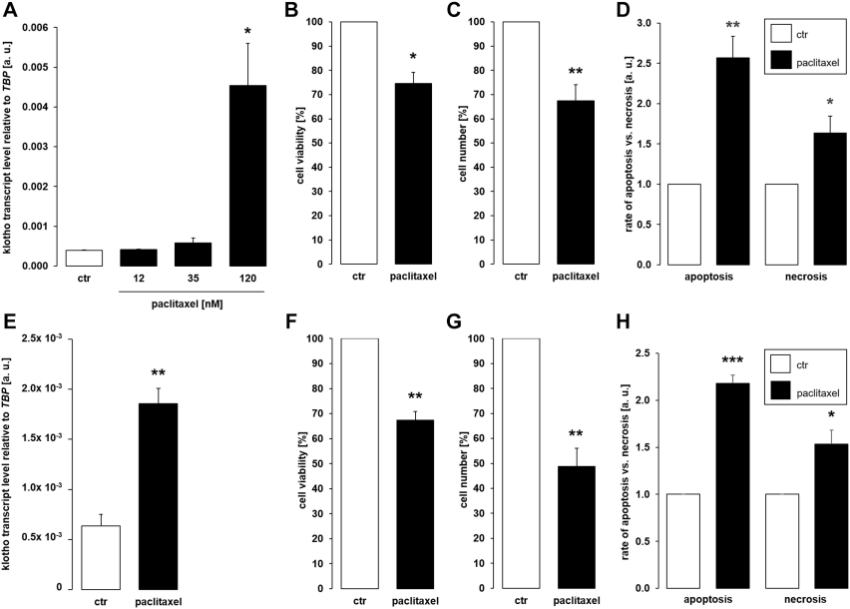
**Paclitaxel induces αklotho expression in MDCK and NRK-52E cells.** (**A**) Arithmetic mean ± SEM of αklotho transcript levels normalized to *TBP* in MDCK cells treated with paclitaxel at the indicated concentration for 24 h (*n* = 5; *Friedman ANOVA* and *Dunn-Bonferroni* post-hoc test). (**B**, **C**) Arithmetic mean ± SEM of MDCK cell viability (**B**) or number (**C**) upon treatment without or with 120 nM paclitaxel for 24 h (**B**: *n* = 4, *one-sample t-*test; **C**: *n* = 5, *one-sample t*-test). (**D**) Rate of apoptosis and necrosis of MDCK cells treated with 120 nM paclitaxel or vehicle control for 24 h (*n* = 6, *one-sample t* test). (**E**) Arithmetic mean ± SEM of αklotho transcript levels relative to *TBP* in NRK-52E cells incubated without or with 120 nM paclitaxel for 24 h (*n* = 5, *paired t-*test). (**F**, **G**) Arithmetic mean ± SEM of NRK-52E cell viability (**F**) or number (**G**) upon treatment without or with 120 nM paclitaxel for 24 h (**F**: *n* = 5, *one-sample t*-test; **G**: *n* = 5, *one-sample t*-test). (**H**) Rate of apoptosis and necrosis of NRK-52E cells treated with or without 120 μM paclitaxel for 24 h (*n* = 5, *one-sample t* test). ^*^p < 0.05, ^**^p < 0.01, ^***^p < 0.001 indicate significant difference from vehicle control; Abbreviations: a. u.: arbitrary units; ctr: control.

Further experiments were performed to elucidate whether cytostatic compound paclitaxel also affects αklotho. To this end, MDCK cells were incubated with different concentrations of paclitaxel for 24 h or with vehicle control, respectively. It is shown in Figure 2A that 120 nM paclitaxel significantly stimulated the abundance of αklotho mRNA. By the same token, 120 nM paclitaxel significantly lowered the viability (Figure 2B) and proliferation (Figure 2C) of MDCK cells. These effects were paralleled by enhanced apoptosis and necrosis (Figure 2D). We also studied the effect of 120 nM paclitaxel in NRK-52E cells. This concentration of the antimitotic agent significantly up-regulated αklotho gene expression within 24 h (Figure 2E), too, whilst down-regulating viability (Figure 2F) and proliferation (Figure 2G) of NRK-52E cells. Similar to MDCK cells, paclitaxel induced apoptosis and necrosis in NRK-52E cells (Figure 2H).

**Figure 3:**
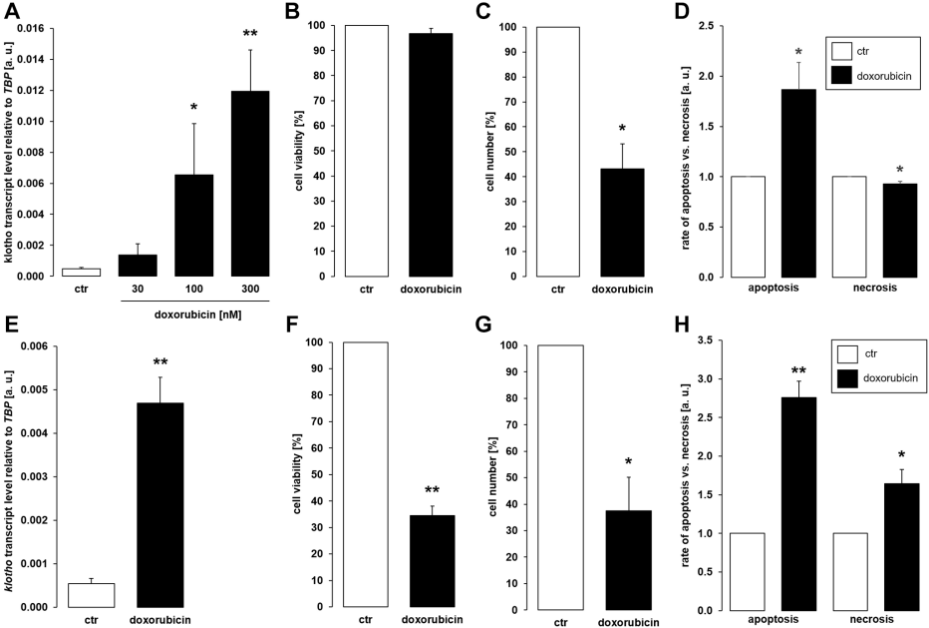
**Doxorubicin enhances αklotho expression in MDCK and NRK-52E cells.** (**A**) Arithmetic mean ± SEM of αklotho transcript levels normalized to *TBP* in MDCK cells treated with doxorubicin at the indicated concentration for 24 h (*n* = 5; *Friedman ANOVA* followed by *Dunn-Bonferroni post-hoc* test). (**B**, **C**) Arithmetic mean ± SEM of MDCK cell viability (**B**) or number (**C**) upon treatment without or with 300 nM doxorubicin for 24 h (**B**: *n* = 5; *one-sample t*-test; **C**: *n* = 4; *one-sample t-*test). (**D**) Rate of apoptosis and necrosis of MDCK cells treated with or without 300 nM doxorubicin for 24 h (*n* = 6, *one-sample t* test). (**E**) Arithmetic mean ± SEM of αklotho transcript levels relative to *TBP* in NRK-52E cells incubated without or with 300 nM doxorubicin for 24 h (*n* = 5, *paired t-*test). (**F**, **G**) Arithmetic mean ± SEM of NRK-52E cell viability (**F**) or number (**G**) upon treatment without or with 300 nM doxorubicin for 24 h (**F**: *n* = 4, *one-sample t*-test; **G**: *n* = 4, *one-sample t*-test). (**H**) Rate of apoptosis and necrosis of NRK-52E cells treated with or without 300 nM doxorubicin for 24 h (*n* = 5, *one-sample t* test) ^*^p < 0.05, ^**^p < 0.01 indicate significant difference from vehicle control; Abbreviations: a. u.: arbitrary units; ctr: control.

As a third common antineoplastic drug, we tested anthracycline doxorubicin. A 24 h-exposure to 100 nM or 300 nM doxorubicin led to a significant increase in the abundance of αklotho transcripts in MDCK cells (Figure 3A). Doxorubicin treatment (300 nM) did not significantly affect viability (Figure 3B) but reduced proliferation (Figure 3C) of MDCK cells. Doxorubicin induced apoptosis while slightly reducing the number of necrotic cells (Figure 3D). In NRK-52E cells, 300 nM doxorubicin readily stimulated αklotho expression within 24 h (Figure 3E) and compromised cell viability (Figure 3F) as well as proliferation (Figure 3G). Apoptosis and necrosis were enhanced by doxorubicin in NRK52-E cells (Figure 3H).

**Figure 4:**
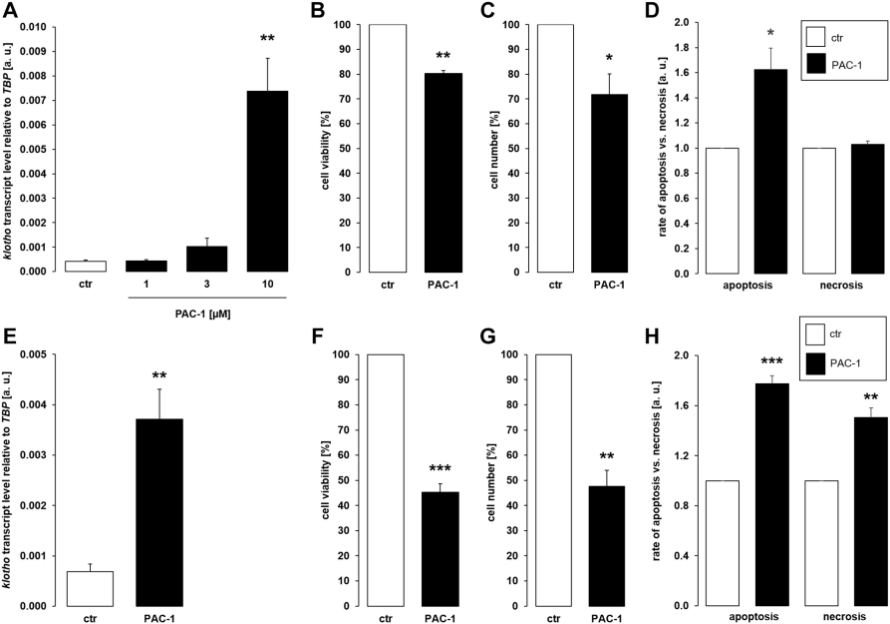
**αKlotho gene expression is stimulated by procaspase activating compound 1 (PAC-1) in MDCK and NRK-52E cells.** (**A**) Arithmetic mean ± SEM of αklotho transcript levels normalized to *TBP* in MDCK cells treated with PAC-1 at the indicated concentration for 24 h (*n* = 6; *Friedman ANOVA* followed by *Dunn-Bonferroni* post hoc test). (**B**, **C**) Arithmetic mean ± SEM of MDCK cell viability (**B**) or number (**C**) upon treatment without or with 10 μM PAC-1 for 24 h (**B**: *n* = 4, *one-sample t*-test; **C**: *n* = 6, *one-sample t*-test). (**D**) Rate of apoptosis and necrosis of MDCK cells treated with or without 10 μM PAC-1 for 24 h (*n* = 6, *one-sample t* test). (**E**) Arithmetic mean ± SEM of αklotho transcript levels relative to *TBP* in NRK-52E cells incubated without or with 10 μM PAC-1 for 24 h (*n* = 6, *paired t-*test). (**F**, **G**) Arithmetic mean ± SEM of NRK-52E cell viability (**F**) or number (**G**) upon treatment without or with 10 μM PAC-1 for 24 h (**F**: *n* = 5, *one-sample t*-test; **G**: *n* = 4, *one-sample t*-test). (**H**) Rate of apoptosis and necrosis of NRK-52E cells treated with or without 10 μM PAC-1 for 24 h (*n* = 5, *one-sample t* test). ^*^p < 0.05, ^**^p < 0.01, ^***^p < 0.001 indicate significant difference from vehicle control; Abbreviations: a. u.: arbitrary units; ctr: control.

Since different classes of cytostatic drugs with pro-apoptotic properties similarly enhanced αklotho expression in MDCK and NRK-52E cells within 24 h, we sought to explore whether direct apoptosis induction also affects αklotho. To this end, we treated the cells with and without caspase 3 activator PAC-1 for 24 h. As demonstrated in Figure 4A, 10 μM PAC-1 induced αklotho expression in MDCK cells, an effect paralleled by decreased cell viability (Figure 4B) and proliferation (Figure 4C). PAC-1 enhanced apoptosis without significantly modifying necrosis (Figure 4D). Also in NRK-52E cells, PAC-1 treatment (10 μM) resulted in a significant surge in αklotho transcripts within 24 h (Figure 4E) and decreased their viability (Figure 4F) and proliferation (Figure 4G). The rates of apoptosis and necrosis were significantly higher in NRK-52E cells upon exposure to PAC-1 (Figure 4H).

**Figure 5:**
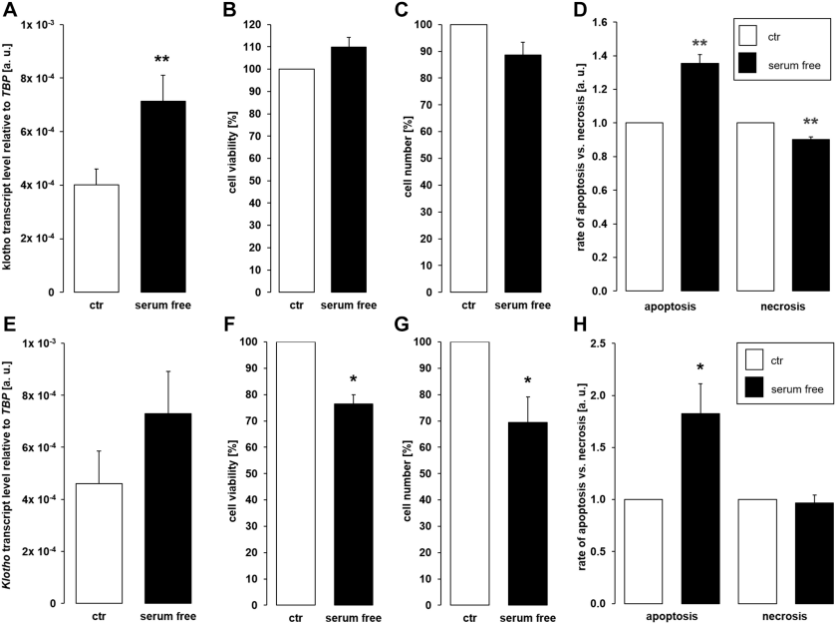
**Serum deprivation up-regulates αklotho expression in MDCK cells.** (**A**) Arithmetic mean ± SEM of αklotho transcript levels relative to *TBP* in MDCK cells incubated for 24 h with or without 5% fetal bovine serum (FBS; *n* = 5; *paired t*-test). (**B**, **C**) Arithmetic mean ± SEM of MDCK cell viability (**B**) or number (**C**) upon incubation with or without 5% FBS for 24 h (**B**: *n* = 4, *one-sample t*-test; **C**: *n* = 6, *one-sample t*-test). (**D**) Rate of apoptosis and necrosis of MDCK cells cultured with or without 5% FBS for 24 h (*n* = 6, *one-sample t* test). (**E**) Arithmetic mean ± SEM of αklotho transcript levels relative to *TBP* in NRK-52E cells incubated for 24 h with or without 5% newborn calf serum (NBCS) (*n* = 8, *paired t-*test). (**F**, **G**) Arithmetic mean ± SEM of NRK-52E cell viability (**F**) or number (**G**) upon incubation with or without 5% NBCS for 24 h (**F**: *n* = 5, *one-sample t*-test; **G**: *n* = 4, *one-sample t*-test). (**H**) Rate of apoptotis and necrosis of NRK-52E cells cultured with or without 5% NBCS for 24 h (*n* = 5, *one-sample Wilcoxon* test). ^*^p < 0.05, ^**^p < 0.01 indicates significant difference from control cells; Abbreviations: a. u.: arbitrary units; ctr: control.

Depriving cells of growth factors through serum depletion similarly favors apoptosis [45]. We therefore aimed to test whether αklotho expression is affected by serum depletion. As depicted in Figure 5A, a 24 h-incubation of MDCK cells in the absence of serum significantly up-regulated αklotho gene expression without significantly impacting on cell viability (Figure 5B) and proliferation (Figure 5C). Serum depletion up-regulated apoptosis whereas necrosis-dependent fluorescence was reduced in serum-starved cells (Figure 5D). In NRK-52E cells, serum depletion did not significantly affect αklotho mRNA levels within 24 h (Figure 5E). However, viability and proliferation were moderately but significantly lower in NRK-52E cells incubated in the absence of serum compared to control cells (Figure 5F, 5G). Serum depletion induced apoptosis and did not significantly affect necrosis in NRK-52E cells (Figure 5H).

**Figure 6:**
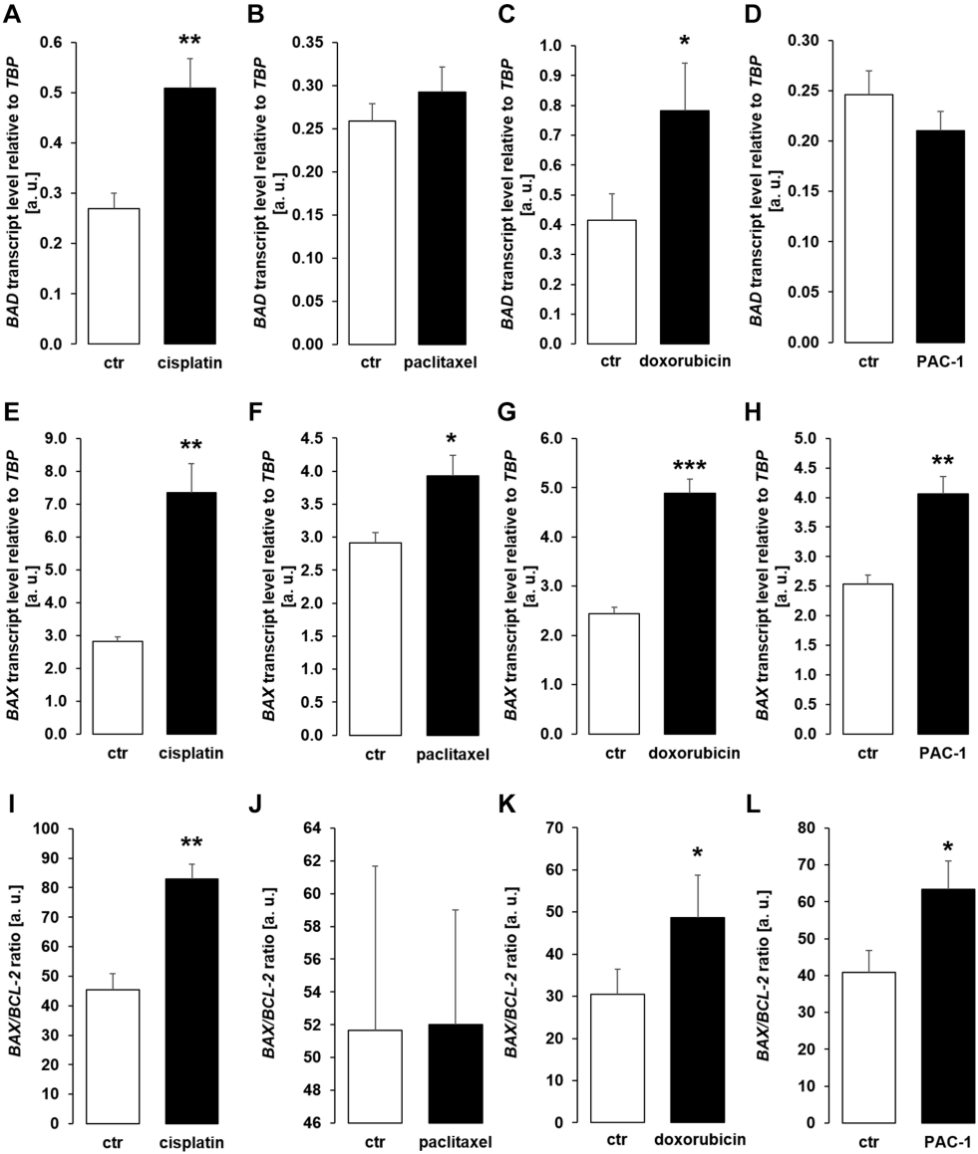
**Cytotoxic agents and PAC-1 up-regulate apoptotic proteins BAD and BAX in MCDK cells.** (**A**–**D**) Arithmetic mean ± SEM of *BAD* transcript levels relative to *TBP* in MDCK cells incubated for 24 h without or with 10 μM cisplatin (**A**; *n* = 5; *paired t*-test), 120 nM paclitaxel (**B**; *n* = 5, *paired t*-test), 300 nM doxorubicin (**C**; *n* = 5, *paired t*-test), or 10 μM PAC-1 (**D**; *n* = 6, *paired t*-test). (**E**–**H**) Arithmetic mean ± SEM of BAX transcripts relative to TBP in MDCK cells treated without or with 10 μM cisplatin (**E**; *n* = 5, *Wilcoxon signed-rank* test), 120 nM paclitaxel (**F**; *n* = 5, *paired t*-test), 300 nM doxorubicin (**G**; *n* = 5, *Wilcoxon signed-rank* test), or 10 μM PAC-1 (**H**; *n* = 6, *paired t*-test). (**I**–**L**) Arithmetic mean ± SEM of BAX to BCL-2 mRNA ratio in MDCK cells incubated for 24 h without or with 10 μM cisplatin (**I**; *n* = 5, *paired t*-test), 120 nM paclitaxel (**J**; *n* = 5, *paired t*-test), 300 nM doxorubicin (**K**; *n* = 5, *paired* t-test), or 10 μM PAC-1 (**L**; *n* = 6, *paired t*-test). ^*^p < 0.05, ^**^p < 0.01, ^***^p < 0.001 indicate significant difference from vehicle control; Abbreviations: a. u.: arbitrary units; ctr: control.

Next, we analyzed gene expression of pro-apoptotic molecules BAD, BAX, and the ratio of BAX/BCL-2 expression in MDCK cells. As illustrated in Figure 6, treatment with cisplatin (Figure 6A, 6E, 6I) or doxorubicin (Figure 6C, 6G, 6K) up-regulated BAD, BAX and BAX/BCL-2 expression. Paclitaxel induced up-regulation of BAX, but did not significantly modify BAD and BAX/BCL-2 (Figure 6B, 6F, 6J) whilst PAC-1 significantly enhanced expression of BAX and BAX/BCL-2, but did not significantly change BAD expression (Figure 6D, 6H, 6L).

**Figure 7:**
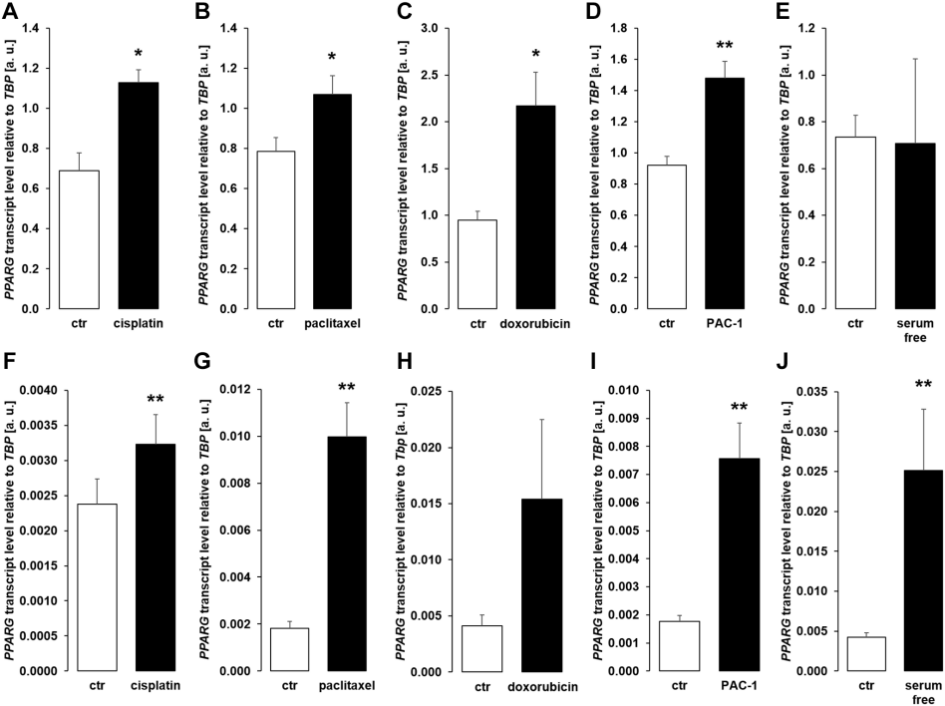
**Cytotoxic agents and apoptosis inducers up-regulate *PPARG* in MDCK and NRK-52E cells.** (**A**–**E**) Arithmetic mean ± SEM of *PPARG* transcript levels normalized to *TBP* in MDCK cells treated with or without 10 μM cisplatin (**A**; *n* = 5; *paired t-*test), 120 nM paclitaxel (**B**; *n* = 5; *paired t-*test), 300 nM doxorubicin (**C**; *n* = 5, *paired t-*test), 10 μM PAC-1 (**D**; *n* = 6, *paired t-*test), or with and without 5% FBS in the culture medium (**E**; *n* = 5, *Wilcoxon signed-rank* test) for 24 h. (**F**–**J**) Arithmetic mean ± SEM of *PPARG* mRNA levels relative to *TBP* in NRK-52E cells treated for 24 h with or without 10 μM cisplatin (**F**; *n* = 8; *paired t-*test), 120 nM paclitaxel (**G**; *n* = 5; *paired t-*test), 300 nM doxorubicin (**H**; *n* = 7, *paired t-*test), 10 μM PAC-1 (**I**; *n* = 6, *paired t-*test), or incubated with or without 5% NBCS in the culture medium (**J**; *n* = 6, *Wilcoxon signed-rank* test). ^*^p < 0.05, ^**^p < 0.01 indicate significant difference from vehicle control; Abbreviations: a. u.: arbitrary units; ctr: control.

We performed further experiments to identify the mechanism underlying enhanced αklotho expression in MDCK and NRK-52E cells exposed to chemotherapeutics or apoptosis stimulants. Since transcription factor PPARγ is pivotal for klotho expression [48] and has been demonstrated to be up-regulated by cisplatin [49], we analyzed *PPARG* expression. As a result, treatment with cisplatin (Figure 7A, 7F), paclitaxel (Figure 7B, 7G), doxorubicin (Figure 7C, 7H), and PAC-1 (Figure 7D, 7I) enhanced *PPARG* expression in both, MDCK and NRK52-E cells. Moreover, serum starvation enhanced *PPARG* in NRK-52E (Figure 7J), but not in MDCK cells (Figure 7E).

**Figure 8:**
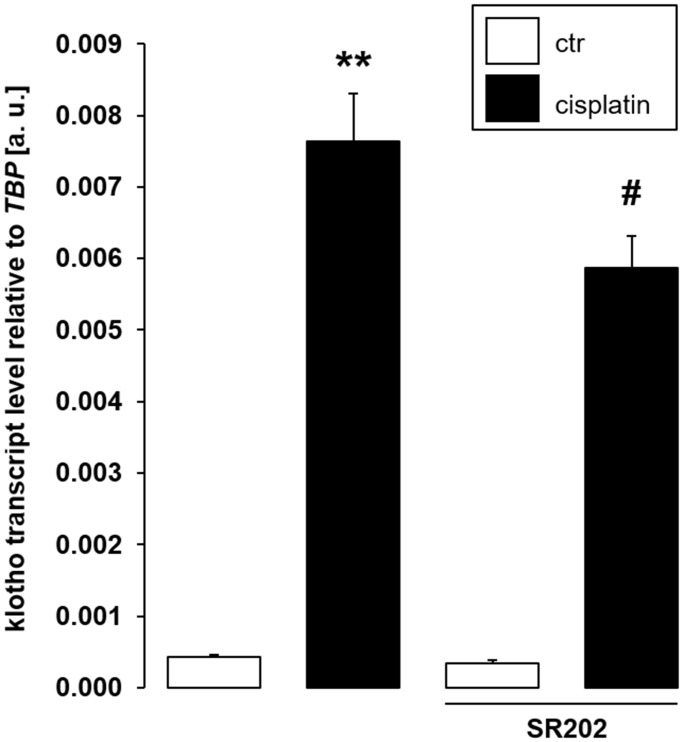
**Selective PPARγ antagonist SR-202 blunts cisplatin-dependent αklotho gene expression in MDCK cells.** Arithmetic mean ± SEM of αklotho transcripts relative to TBP in MDCK cells treated with 3 μM cisplatin or vehicle control in the absence (left bars) or presence (right bars) of 200 μM PPARγ antagonist SR-202 for 24 h (*n* = 8, *repeated measures ANOVA* followed by *Dunnett* post hoc test). ^**^p < 0.01 indicates significant difference from vehicle control (1^st^ bar vs. 2^nd^ bar), ^#^indicates significant difference from the absence of PPARγ inhibitor SR-202 (2^nd^ bar vs. 4^th^ bar); Abbreviations: a. u.: arbitrary units; ctr: control.

In order to confirm that PPARγ is indeed required for cisplatin to up-regulate αklotho expression, we exposed MDCK cells to cisplatin in the presence and absence of PPARγ antagonist SR202. As illustrated in Figure 8, SR-202 significantly blunted cisplatin-dependent up-regulation of αklotho. Hence, PPARγ contributes to enhancement of αklotho expression, but may not fully explain it.

**Figure 9:**
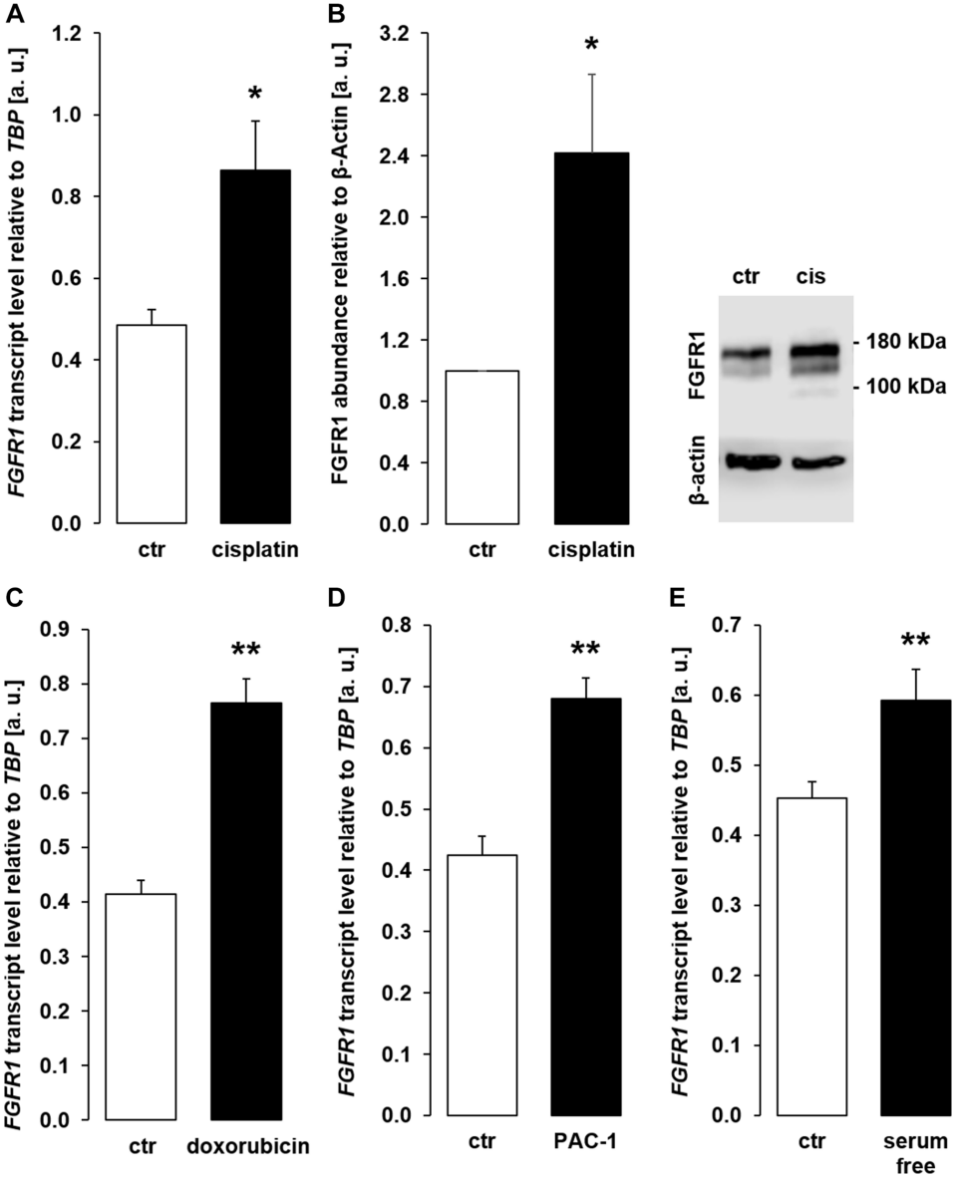
**Cisplatin, doxorubicin, PAC-1, and serum depletion up-regulate FGFR1 in MDCK cells.** (**A**) Arithmetic mean ± SEM of *FGFR1* mRNA levels relative to *TBP* in MDCK cells treated with or without 10 μM cisplatin for 24 h (*n* = 5, *paired t-*test). (**B**) Left panel: Arithmetic mean ± SEM of FGFR1 protein abundance normalized to the abundance of β-actin in MDCK cells following treatment with or without 10 μM cisplatin for 24 h (*n* = 7, *one-sample t*-test). Right panel: Original Western Blot demonstrating the abundance of FGFR1 in MDCK cells treated with (cis) or without (ctr) 10 μM cisplatin for 24 h. (**C**) Arithmetic mean ± SEM of *FGFR1* transcript levels relative to *TBP* in MDCK cells treated with or without 300 nM doxorubicin for 24 h (*n* = 4, *paired t*-test). (**D**) Arithmetic mean ± SEM of *FGFR1* transcript level relative to *TBP* in MDCK cells treated with or without 10 μM PAC-1 for 24 h (*n* = 5, *paired t*-test). (**E**) Arithmetic mean ± SEM of *FGFR1* transcripts relative to *TBP* in MDCK cells incubated without or with 5 % FBS in culture medium for 24 h (*n* = 5, *paired t*-test). ^*^p < 0.05, ^**^p < 0.01 indicate significant difference from vehicle control; Abbreviations: a. u.: arbitrary units; cis cisplatin; ctr: control.

Transmembrane αklotho forms a complex with FGFR1 to serve as a receptor for FGF23. A further series of experiments sought to clarify whether the effect of chemotherapeutics and apoptosis stimulants also affect FGFR1 and/or FGF23 expression in MDCK cells. As demonstrated in Figure 9A, 9B, cisplatin up-regulated FGFR1 expression and protein. Similar effects on FGFR1 expression were observed following incubation with doxorubicin (Figure 9C), PAC-1 (Figure 9D), and upon incubation in serum-free medium (Figure 9E). The expression of FGF23, which is mainly expressed in bone, could not be detected in unstimulated (Ct value: > 40, *n* = 5) MDCK. Cisplatin-treated MDCK cells exhibited lower Ct values for FGF23, however expression was still very low (Ct value: 37.1 ± 1.85, *n* = 5).

ELISA-based quantification of αklotho protein is particularly feasible in human cells. Therefore, we performed further experiments in human proximal tubular cell line HK-2. We treated these cells with the cytotoxic agents and apoptosis stimulants in a way similar to MDCK and NRK-52E cells and measured αklotho transcripts as well as sKL protein by ELISA.

**Figure 10:**
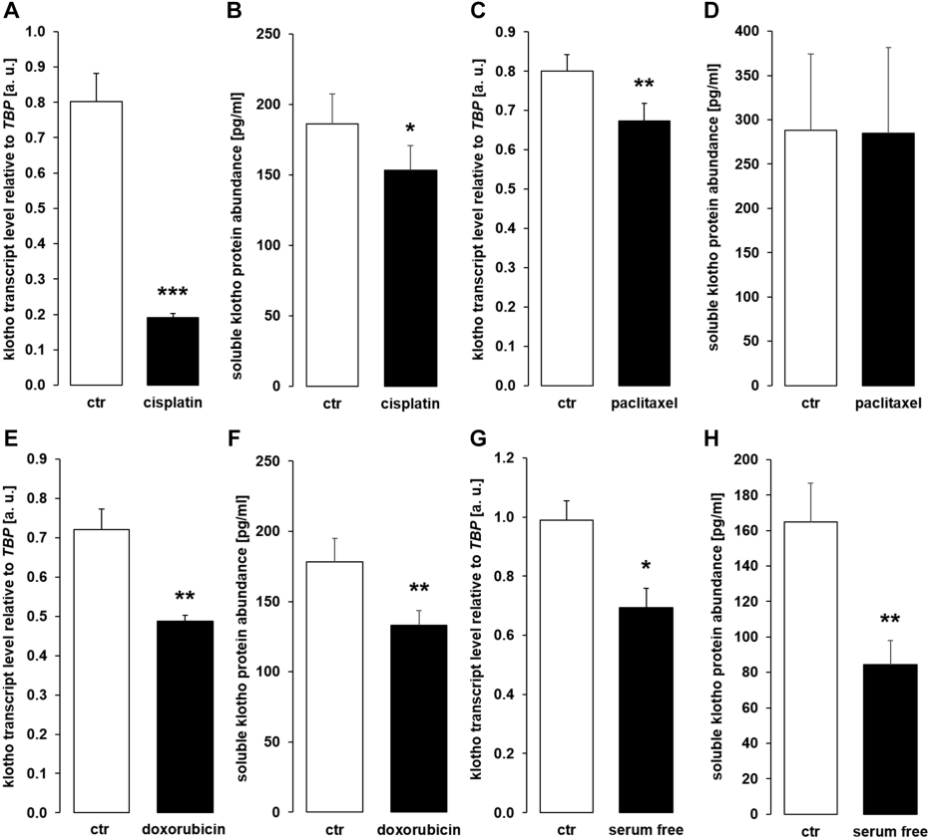
**Cytostatic drugs and serum deprivation reduce αklotho gene expression and soluble klotho (sKL) protein secretion in HK-2 cells.** (**A**) Arithmetic mean ± SEM of αklotho mRNA levels relative to *TBP* in HK-2 cells treated with or without 10 μM cisplatin for 24 h (*n* = 8, *paired t-*test). (**B**) Arithmetic mean ± SEM of sKL concentration in the supernatant of HK-2 cells treated with 10 μM cisplatin or vehicle control for 24 h (*n* = 6, *paired t*-test). (**C**) Arithmetic mean ± SEM of αklotho transcript levels relative to *TBP* in HK-2 cells treated with or without 120 nM paclitaxel (*n* = 6, *paired t*-test) for 24 h. (**D**) Arithmetic mean ± SEM of sKL concentration in the cell culture supernatant of HK-2 cells treated with or without 120 nM paclitaxel for 24 h (*n* = 6, *paired t-*test). (**E**) Arithmetic mean ± SEM of αklotho transcript levels relative to *TBP* in HK-2 cells treated with or without 300 nM doxorubicin for 24 h (*n* = 5, *paired t*-test). (**F**) Arithmetic mean ± SEM of sKL concentration in the cell culture supernatant of HK-2 cells treated with or without 300 nM doxorubicin for 24 h (*n* = 4, *paired t*-test). (**G**) Arithmetic mean ± SEM of αklotho mRNA levels relative to *TBP* in HK-2 cells incubated with (ctr) or without 10 % FBS in the culture medium for 24 h (*n* =5, *paired t*-test). (**H**) Arithmetic mean ± SEM of sKL concentration in the HK-2 cell culture supernatant after incubation with or without 10% FBS for 24 h (*n* = 5, *paired t*-test). ^*^p < 0.05, ^**^p < 0.01, ^***^p < 0.001 indicate significant difference from vehicle control; Abbreviations: a. u.: arbitrary units; ctr: control.

Surprisingly, cisplatin (Figure 10A), paclitaxel (Figure 10C), doxorubicin (Figure 10E), and serum-free incubation (Figure 10G) significantly down-regulated αklotho gene expression. In line with this, sKL protein concentration was lower in the cell culture supernatant of HK-2 cells upon incubation with cisplatin (Figure 10B), doxorubicin (Figure 10F), and in the absence of serum (Figure 10H) and virtually unchanged upon exposure to paclitaxel (Figure 10D).

**Figure 11:**
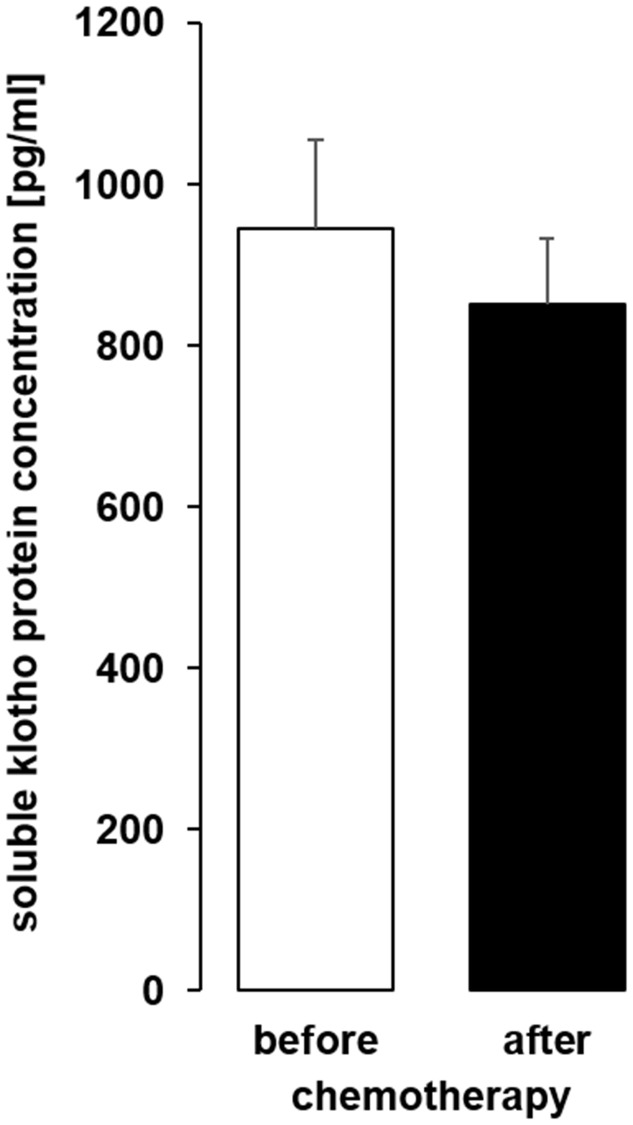
**The serum concentration of soluble klotho (sKL) in patients with cancer before and after administration of a cycle of chemotherapy.** Arithmetic mean ± SEM of sKL serum concentration (*n* = 9; *paired t*-test) in patients 24 ± 4 h before and after administration of a cycle of chemotherapy.

As a last step, we analyzed sKL in serum samples from patients before and after chemotherapy (Table 1) and found that the serum sKL concentration was not significantly different after chemotherapy compared to samples obtained before therapy (Figure 11).

**Table 1.** Patients’ characteristics.

**Patient no.**	**Age**	**Sex**	**Diagnosis**	**Chemotherapy**	**Cycle of chemotherapy**
1	59	m	colon adenocarcinoma	folinic acid, fluorouracil, oxaliplatin, bevacizumab	18
2	62	m	colon carcinoma	folinic acid, fluorouracil, oxaliplatin	2
3	74	m	esophageal carcinoma	fluorouracil, folinic acid, oxaliplatin, docetaxel	4
4	70	m	pancreatic carcinoma	folinic acid, fluorouracil, irinotecan, oxaliplatin	6
5	79	m	esophageal carcinoma	fluorouracil, folinic acid, oxaliplatin, docetaxel	4
6	78	m	esophageal carcinoma	folinic acid, fluorouracil, oxaliplatin	6
7	73	f	pancreatic adenocarcinoma	folinic acid, fluorouracil, irinotecan, oxaliplatin	6
8	61	f	lung carcinoma	nivolumab, ipilimumab, carboplatin, pemetrexed	1
9	80	m	esophageal carcinoma	fluorouracil, folinic acid, oxaliplatin, docetaxel	1

According to our study, αklotho expression was up-regulated by antineoplastic cytostatic agents cisplatin, paclitaxel, and doxorubicin in MDCK and NRK-52E cells within 24 h. Moreover, caspase 3 activator PAC-1 enhanced αklotho expression in both cell lines, whereas serum depletion was only effective in MDCK cells. Caspase 3 activation and serum depletion can be expected to induce apoptosis [44, 45]. In sharp contrast, the same treatment resulted in down-regulation of both, αklotho transcripts and sKL protein, in HK-2 cells. The serum concentration of sKL was not significantly affected by chemotherapy.

Treatment with antineoplastic agents induces cellular stress through different mechanisms: Cisplatin impairs DNA replication by enabling inter- and intrastrand crosslink adducts [41], anthracycline derivative doxorubicin is a topoisomerase II inhibitor and DNA intercalator [42], and paclitaxel is an antimitotic agent that prevents spindle assembly by interacting with tubulin [50]. Ultimately, the cellular impairments induced by these drugs may result in apoptotic cell death, a consequence intended in therapeutic use of these agents in the treatment of different types of cancer [51]. In line with this, cisplatin, doxorubicin, and paclitaxel reduced viability and proliferation of MDCK and NRK-52E cells, albeit to a variable extent. Moreover, the treatment was followed by induction of apoptosis and partially by secondary necrosis. In these two cell lines, apoptosis was paralleled by a marked upregulation of αklotho gene expression. In addition, expression of pro-apoptotic genes BAD, BAX, and BAX/BCL-2 ratio was induced by the chemotherapeutic agents, albeit to a variable extent. Also, direct induction of apoptotic cell death in the absence of cytotoxic drugs up-regulated αklotho mRNA levels in MDCK and NRK-52E cells. According to these results, αklotho expression was upregulated in injured and potentially moribund MDCK and NRK-52E cells prior to their putative death.

Several of the effects of αklotho on major intracellular signaling pathways can be expected to be pro-apoptotic: Inhibition of IGF-1 and insulin signaling [52] as well as Wnt signaling [53]. Also αklotho’s role as a tumor suppressor fits to the concept of αklotho being pro-apoptotic [52]. Accordingly, our findings, i.e., up-regulation of αklotho in MDCK and NRK-52E cells prone to death, may be a novel aspect of the cellular machinery which is part of the initiation and/or execution of apoptosis. Other effects of αklotho including increased anti-oxidant resistance [54], further anti-apoptotic properties [55], or reduced inflammation [29] may rather be associated with being pro-survival. In view of the latter aspect of αklotho signaling, up-regulation of αklotho in damaged and/or dying cells as revealed by our study could therefore be interpreted as an attempt to enhance cellular stress resistance and possibly overcome the injury. In line with this, αklotho has been shown to counteract another form of cell death, necroptosis [46]. Definitely, further studies are necessary to decipher the precise role of increased αklotho expression in cells exposed to potentially deadly noxae.

In an attempt to identify the mechanism underlying αklotho up-regulation in MDCK and NRK-52E following exposure to cytotoxic agents or other apoptosis stimulants, we uncovered a role for transcription factor PPARγ. PPARγ has been demonstrated to be relevant for αklotho expression [48] and is upregulated itself by cisplatin [49]. In line with this, we could confirm that the chemotherapeutics up-regulate *PPARG* in both, MDCK and NRK-52E cells. Moreover, using PPARγ antagonist SR-202 we demonstrated that the cisplatin effect on αklotho in MDCK cells is indeed dependent on PPARγ albeit other factors are likely to be involved, too.

In the kidney, transmembrane αklotho forms a complex with FGFR1, yielding the receptor for bone-derived hormone FGF23 [12]. In line with stimulation of αklotho expression, the cytotoxic agents and apoptosis inducers also up-regulated FGFR1 in MDCK cells.

While our experiments clearly demonstrated up-regulation of αklotho in apoptotic MDCK and NRK-52E cells and uncovered PPARγ as a factor explaining, at least in part, this effect, a completely different response was found in HK-2 cells: The same treatment down-regulated both, αklotho gene expression and sKL concentration in the cell culture supernatant. Several factors may contribute to this discrepancy: Firstly, HK-2 is a human proximal tubule cell line from normal kidney that has been immortalized with human papilloma virus (HPV 16) E6/E7 genes, and these two genes are part of its genome [56]. In contrast, MDCK and also NRK-52E cells are spontaneously immortalized cells [57]. As a matter of fact, E6 and E7 genes used to immortalize HK-2 cells render them more resistant to apoptotic stimuli [58], an effect that may help explain the different response of HK-2 cells observed in our study. Secondly, it also appears possible that the origin of the cells (MDCK cells: dog, NRK-52E: rat, HK-2: human) also contributes to the different response [59]. Thirdly, the renal localization of αklotho may play a role: It is expressed in proximal and, at a higher level, in distal tubule. Renal phosphate handling mainly occurs in the proximal tubule, but its regulation is more dependent on αklotho in the distal tubule [60, 61]. MDCK cells are from distal tubule [62], whereas NRK-52E cells are from proximal tubule [63] as are HK-2 cells [64]. Therefore, the different origin of the cell lines may also contribute to the contrasting results. Moreover, it has to be kept in mind that renal cell lines are only models that do not reflect all aspects of kidney physiology [65]. Therefore, our diverging results using the three different kidney cell lines also underscores that care must be taken when studying αklotho in cell culture.

In a pilot human study, we studied the impact of one cycle of chemotherapy on serum sKL in patients suffering from different types of cancer. We did not observe a significant change of sKL after chemotherapy. It is a major limitation of this small pilot study that patients with different forms of cancer, different chemotherapeutic regimens and different treatment cycles were included. Hence, several aspects may be relevant for our finding: Different forms of cancer themselves impact on αklotho [66]. Moreover, the disease stage and also the number of chemotherapy cycles may influence the effect on αklotho. Although distal tubule is thought to be the main source of sKL [61], also proximal tubule may produce sKL. Given the different response of distal tubular MDCK and proximal tubular HK-2 cells to chemotherapeutics, it appears to be possible that divergent effects also play a role in the human kidney. Definitely, further human studies are warranted to define possible effects of cytotoxic agents on sKL.

Since αklotho plays a particular role in patients with severe disease (e.g., CKD patients [67]), it would of course be of high clinical interest to know whether different responses of αklotho to chemotherapeutics are of clinical relevance and may reflect a different response to the treatment. This should be addressed in further studies.

In conclusion, our study shows that the expression of αklotho gene is stimulated in MDCK or NRK-52E cells exposed to cytotoxic chemotherapeutics cisplatin, doxorubicin or paclitaxel or treated with apoptosis inducers PAC-1 or serum depletion. The effect is, at least in part, dependent on PPARγ. In contrast, the same treatment down-regulates αklotho gene expression and sKL protein in HK-2 cells.

## MATERIALS AND METHODS

### Cell culture

Madin-Darby Canine Kidney cells (MDCK; CCL-34, ATCC, Manassas, VA, USA) were cultured at 37°C and 5% CO_2_ in Dulbecco’s Modified Eagle Medium: Nutrient Mixture F-12 (DMEM/F-12; (Gibco, Life Technologies, Darmstadt, Germany) plus 5% fetal bovine serum (FBS; Gibco), 1% glutamine, and 100 U/mL penicillin and 100 μg/mL streptomycin (Gibco). NRK-52E (CRL-1571, ATCC) cells were cultured in DMEM (Gibco) with 5% newborn calf serum (NBCS; Gibco), 100 U/mL penicillin, and 100 μg/mL streptomycin (Gibco) at 37°C and 5% CO_2_. Human HK-2 cells (CRL-2190, ATCC) were cultured in DMEM with 10% FBS, 100 U/mL penicillin, and 100 μg/mL streptomycin at 37°C and 5% CO_2_. For the experiments, cells were first seeded into 6-well plates (Greiner Bio-One, Frickenhausen, Germany) for 24 h. Subsequently, cisplatin, PAC-1, doxorubicin (all from Tocris Bioscience, Bristol, UK), or paclitaxel (MP Biomedicals, Eschwege, Germany) were added for 24 h as indicated. For serum starvation, culture medium was replaced by serum free medium. After 24 h, cells were either trypsinated and counted with a Neubauer hemocytometer or analyzed for RNA isolation. Selective PPARγ inhibitor SR-202 (Biomol, Hamburg, Germany) was added to the culture medium along with cisplatin at 200 μM. Cell culture supernatants were collected and frozen for further use.

### Quantitative real time PCR

RNA isolation was accomplished by means of RNA-Solv reagent (Omega Bio-Tek, Norcross, GA, USA). For cDNA synthesis 1.2 μg of total RNA was transcribed with the GoScript Reverse Transcription System and random primers (Promega, Mannheim, Germany). Quantitative real time PCR (qRT-PCR) using 2 μl of total cDNA was performed in reaction mixes containing 0.25 μM (αklotho) and 0.5 μM (TATA-binding protein, TBP) of each primer, 10 μl GoTaq qPCR Master Mix (Promega), and sterile water.

The primers used in qPCR analysis are provided in Table 2. αklotho, *PPARG, FGFR1, BAD, BAX, and BCL-2* mRNA levels were normalized to *TBP* mRNA.

**Table 2.** Primers.

**Gene**	**Species**	**Primer sequence (5′ → 3′)**
*klotho*	dog	AAATGAAGCTCTGAAAGCC and AATGATAGAGGCCAAACTTC
*TBP*	dog	CCTATTACCCCTGCCACACC and GCTCCCGTACACACCATCTT
*klotho*	rat	CAACTACATTCAAGTGGACC and CAGTAAGGTTTTCTCTTCTTGG
*TBP*	rat	ACTCCTGCCACACCAGCC and GGTCAAGTTTACAGCCAAGATTCA
*klotho*	human	TGGAAACCTTAAAAGCCATCAAGC and CCACGCCTGATGCTGTAACC
*TBP*	human	TGCACAGGAGCCAAGAGTGAA and CACATCACAGCTCCCCACCA
*PPARG*	dog	CCTCACGAAGAGCCTTCCAA and CCGGAAGAAGCCCTTGCAT
*PPARG*	rat	GAAGCTGTGAACCACTAATATCCA and GCTCTTGTGAACGGGATGTCT
*FGFR1*	dog	AGACAGGTAACAGTGTCGGC and ACGGTTGGGTTTGTCCTTGT
*BAD*	dog	CCAGTGAGCAGGAAGACTCC and TTCCTTCATCCTCGTCGGTC
*BAX*	dog	GATGGCAACTTCAACTGGGG and AAGCACTCCAGCCACAAAGA
*BCL-2*	dog	GGTGAACTGGGGGAGGATTG and TCAAACAGAGGCTGCATGGT

### Viability assay (MTT assay)

Cells were seeded into 96-well plates and treated as described for 24 h and for another hour with 3-[4,5-dimethylthiazol-2-yl]-2,5 diphenyl tetrazolium bromide (MTT; Sigma-Aldrich, Schnelldorf, Germany). Thereafter, the MTT solution was replaced by dimethyl sulfoxide (DMSO; AppliChem, Darmstadt, Germany), and absorption was measured at 550 nm and 690 nm (reference) on a FluoStar Omega plate reader (BMG Labtech, Ortenberg, Germany). Results were normalized to vehicle-treated cells and are given as percentage of viable cells.

### ELISA

HK-2 supernatants and patients’ serum samples were subjected to ELISA for measurement of soluble αklotho protein according to the manufacturer’s protocol (IBL, Hamburg, Germany).

### Apoptosis and necrosis assay

The rate of apoptosis and necrosis was measured using the RealTime-Glo Annexin V Apoptosis and Necrosis Assay (Promega) according to the manufacturer’s protocol.

### Western blotting

MDCK cells were cultured in T25 cell culture flasks (Greiner Bio-One) for 24 h under standard conditions, then incubated with or without 10 μM cisplatin for another 24 h. After cell lysis using RIPA buffer (Cell Signaling Technology, Frankfurt, Germany) supplemented with protease and phosphatase inhibitor cocktail and EDTA (Halt, Thermo Scientific), total protein concentration was measured by Bradford assay (Bio-Rad). Thirty μg of total protein were subjected to standard 10% SDS-PAGE and Western Blotting. The following antibodies were used: anti-FGF receptor 1 (D8E4), anti-β-actin (8H10D10), anti-rabbit IgG HRP-linked (all from Cell Signaling Technology), and anti-mouse IgG HRP-linked antibody (Abcam, Cambridge, UK). For visualization, membranes were incubated for 2 min with Westar Nova 2.0 (β-actin) or Westar Supernova (FGFR1) ECL substrate (both from Cyanagen, Bologna, Italy). Densitometrical analysis was performed on a C-Digit^®^ Blot scanner (Li-Cor, Lincoln, NE, USA) and FGFR1 bands were normalized to β-actin bands using the Image Studio™ software (Li-Cor).

### Patients

Serum samples were collected from cancer patients of the Department of Oncology, University Hospital of Martin-Luther-University Halle-Wittenberg, Halle (Saale), Germany. The study was approved by the ethics committee of Martin-Luther-University (approval no. 2014–75). Blood samples were collected 20 ± 4 h before and after chemotherapy, centrifuged and frozen at −70°C until analysis. Patient characteristics are depicted in Table 1.

### Statistics

Data represent arithmetic mean ± standard error of the mean (SEM) with n denoting the number of independent experiments. Groups were tested for normal distribution using Shapiro-Wilk test. The cell number and viability experiments were analyzed with one-sample *t*-test or alternatively with one-sample Wilcoxon signed rank test, as appropriate. Data with more than two groups were analyzed with repeated measures analysis of variance (ANOVA) followed by Dunnett’s multiple comparison test or with non-parametric Friedman ANOVA and Dunn-Bonferroni post-hoc test. If *p* < 0.05, differences were considered significant. SPSS software was used for statistical data evaluation (IBM Version 27.0; Armonk, NY, USA).
